# Nickel on Oxidatively Modified Carbon as a Promising Cost-Efficient Catalyst for Reduction of *P*-Nitrophenol

**DOI:** 10.3390/molecules27175637

**Published:** 2022-09-01

**Authors:** Shamil Galyaltdinov, Anna Svalova, Vasiliy Brusko, Maria Kirsanova, Ayrat M. Dimiev

**Affiliations:** 1 Laboratory for Advanced Carbon Nanomaterials, Chemical Institute, Kazan Federal University, Kremlyovskaya Str. 18, 420008 Kazan, Russia; 2 Advanced Imaging Core Facility, Skolkovo Institute of Science and Technology, 121205 Moscow, Russia

**Keywords:** oxidatively modified carbon, single-atom catalysis, *p*-nitrophenol reduction, nickel, UV-spectroscopy

## Abstract

The reduction of *p*-nitrophenol to *p*-aminophenol has become a benchmark reaction for testing the efficiency of new catalytic systems. In this study, we use oxidatively modified carbon (OMC) as a structural support to develop a new cost-efficient nickel-based catalytic system. The newly developed material comprises single nickel ions, chemically bound to the oxygen functional groups on the OMC surface. The highly oxidized character of OMC ensures the high lateral density of nickel ions on its surface at relatively low nickel content. We demonstrate excellent catalytic properties of the new material by using it as a stationary phase in a prototype of a continuous flow reactor: the reagent fed into the reactor is *p*-nitrophenol, and the product, exiting the reactor, is the fully converted *p*-aminophenol. The catalytic properties of the new catalyst are associated with its specific morphology, and with high lateral density of active sites on the surface. The reaction can be considered as an example of single-atom catalysis. The resulting material can be used as an inexpensive but efficient catalyst for industrial wastewater treatment. The study opens the doors for the synthesis of a new series of catalytic systems comprising transition metal atoms on the OMC structural support.

## 1. Introduction

One of the main environmental pollutants, *p*-nitrophenol, is originated in large quantities as the waste of the pharmaceutical industry, in the manufacture of organophosphorus pesticides and their decomposition products [1,2,3]. *P*-nitrophenol is a toxic substance that can penetrate the human body, cause damage to organs, such as kidneys, lungs, and have a harmful influence on the central nervous system, not to mention the poisoning of aquatic life [2,4,5]. To prevent the negative environmental impact of *p*-nitrophenol, it can be reduced to *p*-aminophenol, the toxicity of which is about 500 times lower [6]. In addition, *p*-aminophenol can be used as a photographic developer, corrosion inhibitor, anticorrosion lubricant, hair-dyeing agent, etc. [7,8,9,10]. Since the reduction of *p*-nitrophenol without a catalyst does not occur due to the high kinetic barrier [11], there is constant interest in the design of efficient and inexpensive catalysts that would help to purify industrial wastewater. In recent years, the reduction of *p*-nitrophenol has become a benchmark reaction for testing various novel catalytic systems for the simplicity of monitoring the reaction progress [9,12].

In the majority of papers, devoted to the reduction of *p*-nitrophenol, the developed catalysts are based on noble metals, such as Au, Pd, Pt, Ru, and Ag in the form of nanoparticles [13,14,15,16,17,18,19]. However, the high cost of noble metals, their limited availability, and the ever-growing demand stimulate the search for cheaper and more available alternatives. Nickel-based materials [17,19,20,21], which are noticeably cheaper than noble metals, can be considered as an alternative. Interestingly, most publications, devoted to the development of nickel-based catalysts, pursue the targeted fabrication of nickel-containing nanoparticles, usually involving multi-step procedures.

Graphene oxide (GO) is a promising substrate for designing the metal on graphene support catalysts, because it can bind various metal cations in aqueous media [22,23,24,25]. In a series of our recent publications, we have demonstrated the possibility of coordinating several metal cations such as Gd^3+^, Fe^3+^, Mn^2+^, Sr^2+^, Cs^+^, etc., with GO in aqueous solutions [26,27]. Based on this property of GO, in our previous work [12], we synthesized a new catalytic system, comprising individual Ni atoms-ions on reduced GO support, which demonstrated remarkable results in the reaction of reducing *p*-nitrophenol. Importantly, nickel on the RGO surface existed in the form of single atoms-ions, being strongly coordinated by the oxygen groups of GO, due to its specific chemistry.

Despite numerous advantages, GO has one significant shortcoming, namely, its high cost, related to the details of its production. First of all, the oxidation of graphite is slow and proceeds in three consecutive steps [28]. Secondly, the synthesis requires the use of large amounts of aggressive reagents, such as sulfuric acid and potassium permanganate in Hummers’ method [29], or fuming nitric acid and potassium chlorate in Brodie’s method [30,31]. For example, the oxidation of 1 g of graphite requires 3–6 g of potassium permanganate and 120–200 mL of sulfuric acid [28,32,33]. These reagents form huge quantities of byproducts and acidic waste. Thirdly, the bottleneck of the GO synthesis is its purification from sulfuric acid and manganese derivatives, which includes at least 5–6 cycles of dispersion/centrifugation, since GO cannot be separated from acidic waste by other means. This requires significant recourses of electricity and manpower [34].

In a recent publication, we developed a new material, named oxidatively modified carbon (OMC) that was aimed to serve as an inexpensive alternative to GO for removing radionuclides from water [35]. OMC is made by oxidizing bituminous coke and some other carbon sources. The main advantage of OMC over GO is its particulate 3D structure, and thus, the possibility to be used in a column filtration setup, which is impossible with 2D GO sheets, clogging filters, and columns. Since OMC has the same functional groups as GO, it demonstrated a similar sorption capacity toward the two tested ions Cs^+^ and Sr^2+^. At the same time, OMC is significantly cheaper. First of all, preparation of OMC requires only 0.9–1.2 weight equivalents of potassium permanganate and ~20 mL of sulfuric acid per 1 g of carbon source, i.e., four and seven times less compared to the respective quantities in the synthesis of GO. Finally, and most importantly, purification of as-prepared OMC from the acid waste does not require centrifugation, since particulate OMC can be easily separated from acid by decantation and/or filtration. This all makes the cost of the OMC production ~10 times lower compared to that for GO [35]. All these factors together open up the possibility of developing a new material based on OMC and Ni that can potentially serve as a catalyst for various reduction and hydrogenation reactions. This catalyst might be as efficient as GO-Ni, but significantly less expansive.

The aim of this paper is to prepare a new material based on reduced OMC and nickel, and to study its catalytic activity towards the reduction of *p*-nitrophenol to *p*-aminophenol.

## 2. Experimental

### 2.1. Materials

Sulfuric acid (96%, Shchekinoazot LLC, Pervomaiskii industrial community, Tula Oblast, Russia), potassium permanganate (99.5%, MCD Company, Moscow, Russia), sodium tetrahydroborate NaBH_4_, nickel nitrate Ni(NO_3_)_2_ · 6H_2_O (99.5%, TatKhimProduct CJSC, Kazan, Russia), and C–Seal F carbon (MiSWACO company, Houston, TX, USA) were used without further purification.

### 2.2. Synthesis of Oxidized Modified Carbon (OMC)

To prepare OMC, 10 g of the carbon source C-Seal F was dispersed in 200 mL of concentrated sulfuric acid at RT with constant stirring using an overhead stirrer. After 40 min of stirring, 12 g potassium permanganate was added to the mixture in small portions, which corresponds to the 1.2 weight equivalents. The reaction completion was determined by the absence of a red-pink color of Mn(VII) upon diluting a sample of the reaction mixture with water [32]. Then, the reaction was quenched by adding 700 g of an ice-water mixture. After stirring the diluted mixture for 12 h, it was filtered on a Buechner funnel and washed several times with DI water until the filtrate was neutral. The filter cake was dried in open air to the constant weight. As a result, 11.62 g of dry OMC was obtained.

### 2.3. Synthesis of the Reduced OMC/Ni (rOMC/Ni) Composite

A portion of the resulting OMC, 1 g, was dispersed in 35 mL of DI water at RT with constant stirring on a magnetic stirrer. The 10% solution of nickel nitrate (Ni(NO_3_)_2_ · 6H_2_O), which contained 1.86 g of dry salt, was added to this dispersion. The reaction mixture was stirred for 30 min and then filtered through a membrane filter (d = 47 mm, pore size is 0.4 µm). The filtrate had greenish color, indicating that not all the nickel ions were bound to OMC. The filter cake (OMC/Ni^2+^) was redispersed in DI water, and 3 g of solid sodium tetrahydroborate (NaBH_4_) was added to the suspension in small portions as a reducing agent. The completion of the reduction was monitored by the absence of hydrogen bubbles in the mixture. The as-formed reduced product (*r*OMC/Ni), was filtered through a membrane filter and washed with DI water until the filtrate had pH~7. The product was dried in air at RT. As a result, 0.79 g of the dry *r*OMC/Ni was obtained.

For a control experiment without Ni^2+^ ions, 200 mg of the previously obtained OMC was reduced with 4 weight equivalents of sodium tetrahydroborate (800 mg), as described above. After 24 h, the precipitate was filtered off on a membrane filter and washed with DI water, until the filtrate had pH = 7. The filtercake was dried at RT to the constant weight. The sample was named as *r*OMC.

### 2.4. X-ray Photoelectron Spectroscopy (XPS)

The spectra of the *r*OMC/Ni composite were acquired in a Phoibos 100/150 UHV chamber of the multi-technique surface analysis system from SPECS (SPECS Surface Nano Analysis GmbH, 13355 Berlin, Germany). The Mg Kα X-ray source operating at 12.5 kV and 250 W was used. A pass energy of 30 eV (step size of 0.5 eV) was used for wide-range scans; pass energy of 20 eV (step size of 0.1 eV) was used for high-resolution measurements. All spectra were analyzed by using the CasaXPS software (CasaXPS Version 2.3.24PR1.0, Teignmouth, UK).

The *thermogravimetric analysis* (TGA) was performed with an STA 449 F5 Jupiter analyzer from Netzsch (NETZSCH-Gerätebau GmbH, 95100 Selb, Germany) in Ar atmosphere with a total flow rate of 80 mL/min.

The *scanning electron microscopy* (SEM) images were acquired with a Merlin field-emission high-resolution scanning electron microscope from Carl Zeiss (ZEISS, Oberkochen, Germany) at accelerating voltage of incident electrons of 5 kV and current probe of 300 pA.

The samples for *scanning transmission electron microscopy (STEM)* investigation were prepared by deposition of the *r*OMC/Ni suspension onto a Cu grid with supporting Lacey/carbon layer. The high-angle annular dark field scanning transmission electron microscopy (HAADF-STEM) images were taken on a Titan Themis Z microscope equipped with a DCOR+ spherical aberrations corrector and operated at 200 kV. Energy-dispersive X-ray (EDX) spectra and elemental maps were collected in the STEM mode using the Super-X system, consisting of four large-area ring-shaped SSD detectors. The EDX spectra and maps were processed with the Velox software (Velox 2.14, ThermoFisher Scientific, Waltham, MA, USA).

The *UV/Vis spectra* were recorded with Shimadzu UV-2600 spectrophotometer in the optical cell with 1.0 cm light pass at 20 °C.

### 2.5. Catalytic Reduction of P-Nitrophenol

The catalytic activity of the prepared composite was tested in two ways.

*First method.* The catalytic properties of the sample were tested in a prototype of a continuous flow reactor. It was made from a glass pipette (*V* = 10 mL, *d* = 6 mm) that served as a column. The column nose was plugged with cotton, followed by a layer of washed calcined sand, a layer of 0.014 g of *r*OMC/Ni composite, and another top layer of the sand.

First, 20 mL of a mixture of *p*-nitrophenol (*C* = 0.1 mM) with a 50-fold excess of sodium tetrahydroborate (*C* = 5 mM) (**mixture 1**) was passed through the column. The solution passed through the column was collected in portions: the first two portions were 2.5 mL, and all subsequent portions were 5 mL. Then, the column was washed with DI water. After that, another 20 mL of another solution of *p*-nitrophenol (*C* = 1 mM) with the same excess of sodium tetrahydroborate (*C* = 50 mM) was passed through the column (**mixture 2**). The solution passed through the column was collected in several portions of 5 mL.

*Second method.* The catalytic activity of the prepared *r*OMC/Ni was tested upon its constant contact with a solution of *p*-nitrophenol with two different concentrations of *p*-nitrophenol. In the first experiment, 8 mg *r*OMC/Ni was dispersed in 20 mL of 0.1 mM *p*-nitrophenol solution, containing a 100-fold excess of sodium tetrahydroborate (*C* = 10 mM) (**mixture 3**). In the second experiment, 90 mg of *r*OMC/Ni was dispersed in 20 mL of 1 mM *p*-nitrophenol, containing a 100-fold excess of sodium tetrahydroborate (*C* = 100 mM) (**mixture 4**). In addition, a blank experiment was performed separately without Ni^2+^ ions. For this, 90 mg of *r*OMC was dispersed in **mixture 4**. During experiments, the reaction mixture was constantly agitated, using a Vortex shaking device. Portions of solution were sampled out in a certain period of time to monitor the reaction progress.

## 3. Results and Discussion

### 3.1. Characterization

**Figure 1:**
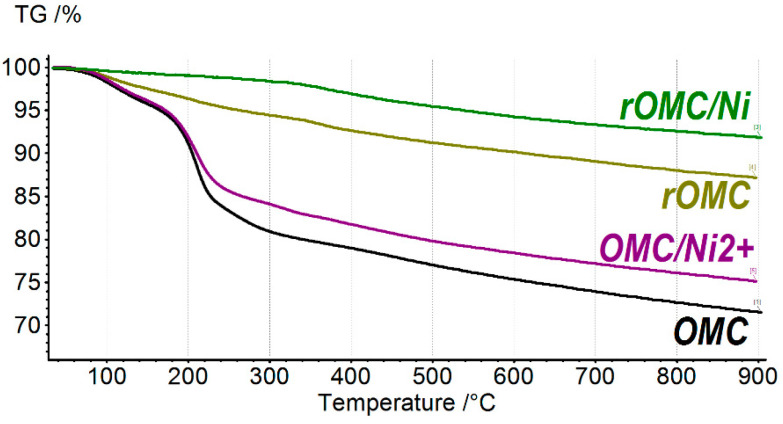
TGA curves for OMC, OMC/Ni^2+^, *r*OMC/Ni, and *r*OMC samples.

Samples of the prepared OMC, OMC/Ni^2+^, *r*OMC/Ni composite, and *r*OMC were characterized by thermal gravimetric analysis. The corresponding thermogravimetric curves are shown in Figure 1.

According to the TGA data, the as-prepared OMC has a decomposition TGA curve, similar to that for GO. The sample has a weight loss of ~2% up to 120 °C, which is related mostly to the loss of adsorbed water. The main weight loss of 19.2% is in the range of 180–300 °C. This is the characteristic weight loss of GO, associated with the decomposition of major oxygen-containing groups, such as epoxy and hydroxyl groups. The similar GO decomposition behavior suggests similar chemical composition. The residual weight is about 71%, which is notably higher than that for GO, which normally loses ~60% of its original weight. This suggests that the overall oxidation degree of OMC is lower than that for GO [32]. The OMC/Ni^2+^ sample has a weight loss of 4% lower than that of pure OMC. The difference of 4% is related to the presence of Ni^2+^ ions in the OMC/Ni^2+^ sample.

The TGA data for *r*OMC/Ni show that the weight loss up to 300 °C is < 2%, which is 10 times less than the weight loss for the original OMC sample in the same temperature range (Figure 1). Such a strong difference indicates that *r*OMC/Ni has a high degree of reduction, and contains only a small number of remaining oxygen-containing groups. In addition, the weight loss up to 120 °C is only ~0.5%, which indicates that water is practically not sorbed by the catalyst due to the hydrophobic nature of the surface. The weight loss above 300 °C can be associated with the decomposition of the remaining oxygen-containing groups, which is also typical for the *r*GO/Ni composite [12]. Most likely, these oxygen-containing groups participated in the coordination of Ni^2+^ ions [12].

The slope of the TGA curve for *r*OMC in the 30–300 °C temperature range is steeper than that for *r*OMC/Ni. This signifies that in the absence of Ni^2+^ ions, the degree of the OMC reduction is lower. Thus, Ni^2+^ ions catalyze the reduction of OMC with sodium tetrahydroborate and promote its deeper reduction.

**Figure 2:**
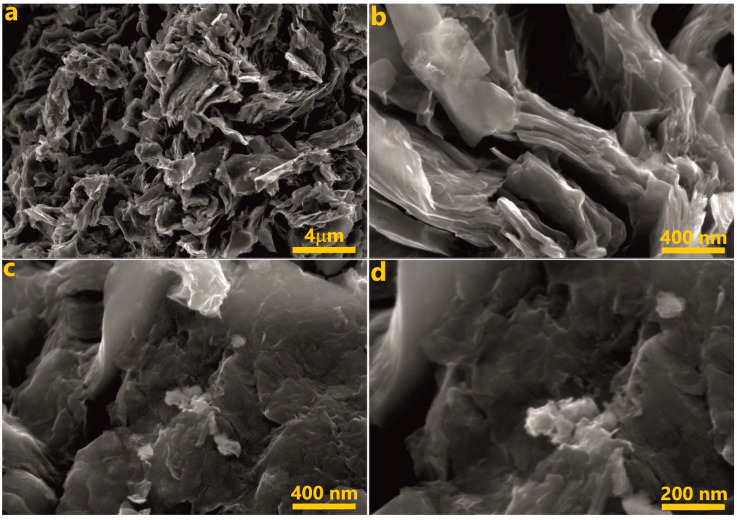
SEM images of *r*OMC/Ni, acquired at different magnifications: (**a**) low magnification image; (**b**–**d**) higher magnification images.

According to the SEM images (Figure 2), the *r*OMC/Ni sample has a layered structure and consists of particles that resemble crumpled paper or rose petals. This pattern is also typical for the original OMC, i.e., the reaction with nickel and subsequent reduction do not notably change the morphology of the material [35]. There are voids between the petals. These voids and the layered structure ensure the high surface area of *r*OMC/Ni, which is an important issue for a heterogeneous catalyst. Importantly, there are no nanoparticles, observable on the OMC surface even at high magnifications (Figure 2b–d).

**Figure 3:**
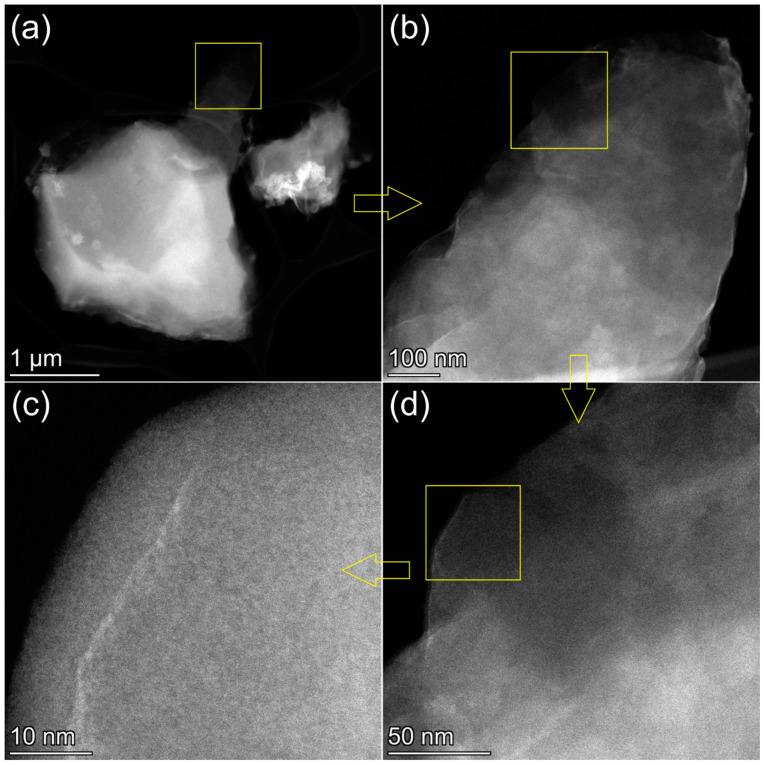
The HAADF-STEM images of an *r*OMC/Ni particle at different magnifications. (**a**) low magnification image; (**b**–**d**) progressively higher magnification images. The yellow squares and arrows indicate the area on the lower magnification image, where the next higher magnification image is acquired.

To further explore the condition of nickel on the *r*OMC surface, we employed the HAADF STEM experiments. As is obvious from the HAADF images, the OMC surface is smooth; there are no observable particles even at the highest magnification (Figure 3).

**Figure 4:**
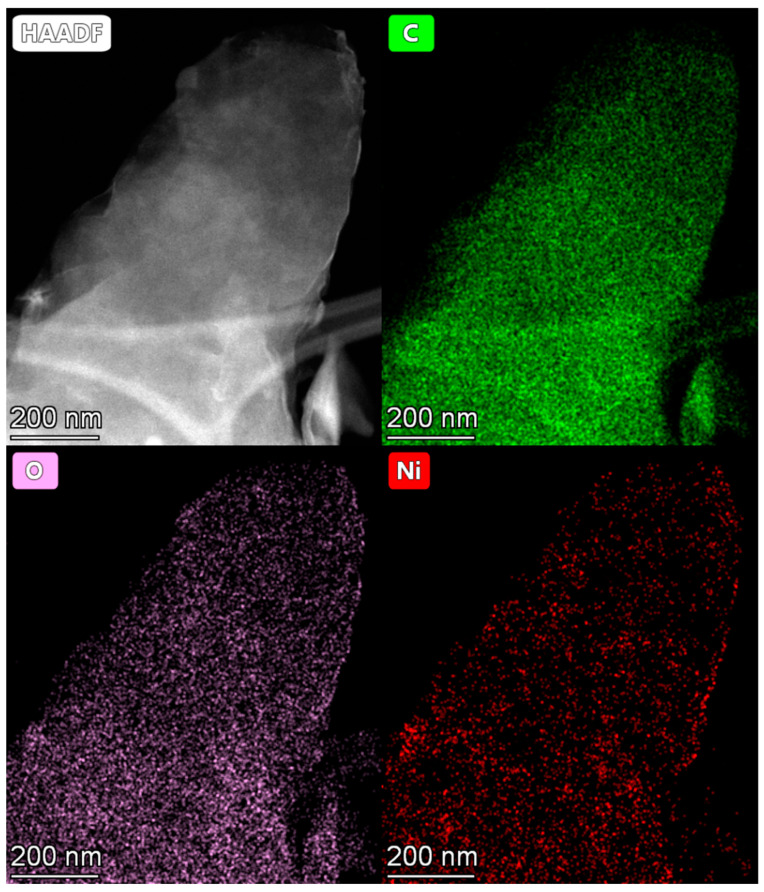
The EDX mapping of the area shown in Figure 3b.

At the same time, the EDX mapping, performed from the area shown in Figure 3b, confirms that nickel is present in the sample in significant quantities, and it is evenly distributed over the observed area, following the patterns for the distribution of carbon and oxygen (Figure 4). The higher the number of the OMC layers in the acquisition area, the stronger the signals for C, O, and Ni. The brighter areas in the low-magnification HAADF-STEM images (Figure 3a,b) are most likely related to the higher number of carbon layers, and, respectively, to the higher content of Ni. Note, acquisition of the images with atomic resolution from amorphous OMC is not possible. However, the quality of the high magnification HAADF-STEM images (Figure S1) is sufficient to resolve single atomic layers of OMC (see left-bottom corner on Figure S1f). This observation additionally confirms that OMC has a graphitized layered structure. However, there is still no particulate matter detectable in the right-upper corner of the same image, exposing the brighter areas on the HAADF images.

The image, acquired with four times higher magnification than that in Figure S1f, also did not reveal any nanoparticles or clusters (Figure S2). Note, even a four–five Ni atom cluster would manifest on such an image as a bright spot. The lack of bright spots suggests that the number of Ni atoms in clusters, if they exist, is fewer than four. Thus, the microscopy analysis suggests that nickel does not form any detectable nanoparticles, and it is evenly distributed over the *r*OMC surface approaching a single or few-atom level morphology. This observation is consistent with the literature data on *r*GO/Ni [12], since GO and OMC have a similar chemical composition in terms of the chemical groups, populating their surfaces.

**Figure 5:**
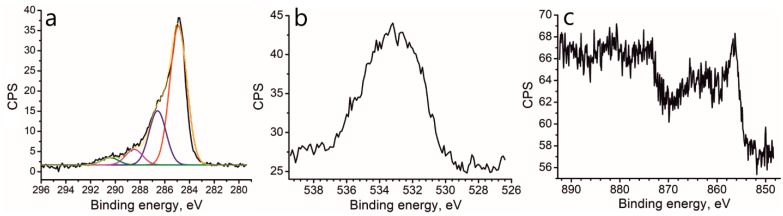
Elemental XPS spectra for the *r*OMC/Ni sample: (**a**) C1s XPS spectrum; (**b**) O1s XPS spectrum; (**c**) Ni2p XPS spectrum.

The C1s XPS spectrum of *r*OMC/Ni contains a single peak at 284.9 eV, which corresponds to the non-oxidized carbon atoms (Figure 5a). A peak shoulder at 288–286 eV indicates the presence of small quantities of the oxidized form of carbon atoms. Deconvolution of the integral spectrum resolves components at 286.6 eV, 288.5 eV, and 290.4 eV, associated, respectively, with tertiary alcohols and epoxides, ketones, and carboxyl groups. The presence of residual oxygen groups is also consistent with a small weight loss according to the TGA data (Figure 1). Apparently, the remaining oxygen atoms, whose signal is well pronounced (Figure 5b), are participated in the coordination of Ni^2+^ ions.

According to the XPS data, the sample contains 87.2 at% C, 12.0 at% O, and 0.8 at% Ni. The Ni2p_3/2_ component in the XPS spectrum is centered at 856.4 eV (Figure 5c). The signal/noise ratio is low due to the low nickel content, but one can still clearly differentiate the main spectrum components. Note, for metallic nickel this component is situated at 852.2 eV. The region at ~856 eV is typical for Ni^2+^ in compounds such as NiO and Ni(OH)_2_. Therefore, one can conclude that nickel remains in the unreduced state, but not in the form of Ni^0^, despite the use of the strong reducing agent sodium tetrahydroborate. Note that the registered binding energy for Ni 856.4 eV is even higher than that for NiO. This observation can be explained by the fact that one Ni^2+^ ion is coordinated simultaneously by two and more oxygen atoms. This observation is consistent with the literature data for the *r*GO/Ni sample [12]. Thus, we can conclude that the coordination of Ni^2+^ with oxygen-containing groups of OMC is quite strong and prevents the nickel ions from reduction.

### 3.2. Catalytic Activity of the rOMC/Ni

**Figure 6:**
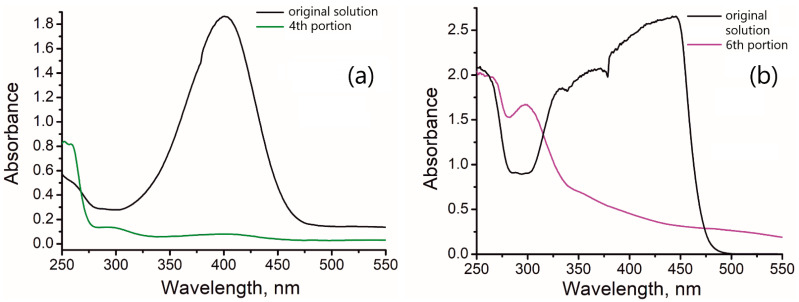
The UV–Vis spectra of original **mixtures 1** (**a**) and **2** (**b**) and corresponding reduction products.

Figure 6 shows UV–Vis spectra of the original **mixtures 1** and **2** and their reduction products after their passage through the column with the catalyst.

The original mixtures of *p*-nitrophenol with a 50-fold excess of sodium tetrahydroborate have an intense yellow color, which is due to the formation of a *p*-nitrophenolate ion in a strongly alkaline medium. The peak at 400 nm in the UV spectrum of the original solutions corresponds to the absorption of the *p*-nitrophenolate ion (Figure 4). **Mixture 2** has an intense absorption signal that goes beyond the measurement range of the instrument (Figure 6b). The solution, passing the column, is absolutely colorless. Therefore, the visual disappearance of the yellow color of the original mixture can serve as an indicator of the reaction progress. It should be noted, however, that the first 2.5 mL of the solution exiting the column is also colorless, but here the disappearance of the color is explained by the sorption of *p*-nitrophenolate ion or/and the reaction product by the catalyst. This is evident from the absence of the *p*-aminophenol signal in UV–Vis for the first portion (Figure S3, SI).

The spectra of the collected portions, exiting the column, beginning the third portion are similar. Only the spectrum for the fourth portion is shown in Figure 6a for simplicity. As evident from Figure 6a, the signal at 400 nm almost completely disappears, and the signal at 300 nm, associated with *p*-aminophenol, appears when the solution passes through the column. When **mixture 2** is passed through the column (Figure 6b), the color of the solution disappears already for the first 5 mL of the solution (Figure S4, SI). This is caused by the reduction of *p*-nitrophenol, since, along with the disappearance of the peak at 400 nm, a pronounced peak of the product at 300 nm appears. The next sequentially passed portions of the solution have an increasingly pronounced peak at 300 nm, which indicates accumulation of the product in the solution. The most intense peak at 300 nm is observed when the sixth portion of the solution is sampled (Figure 6b). Simultaneously, there is no absorbance at 400 nm, suggesting complete elimination of the reactant. Thus, the nearly quantitative conversion of the reactant to the product in the prototype of the continuous flow reactor suggests the efficiency of the newly developed catalyst.

**Figure 7:**
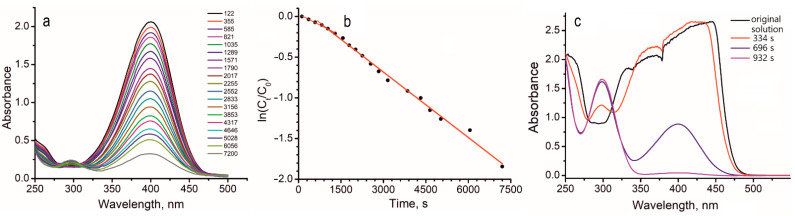
The kinetics measurements: (**a**,**b**) The experiment with **mixture 3**, concentration of *p*-nitrophenol is 0.1 mM. (**a**) The acquired spectra, (**b**) the function of ln(C_t_/C_0_) on time. (**c**) The experiment with **mixture 4**; the spectra for the original solution and the reduction products acquired after 334, 696, and 932 s from the start of the reaction.

Several studies on this reaction include the kinetic measurements, based on registering the change in the 400 nm peak intensity with time. Subsequently, the rate constants and even activation energies were calculated. In our previous study [12], we stressed that the use of this approach toward heterogeneous catalysis is not straightforward. In this case, the measured rate depends mostly on the efficiency of the mass transfer from the bulk of solution to the catalyst surface, but not on the actual rate of the chemical interaction. The mass transfer, in turn, depends on such factors as the size and morphology of the catalyst particles, the content of the catalyst in the reaction mixture, the agitation intensity, etc. Since such measurements toward this reaction are broadly performed in the literature, in this work, we still performed similar kinetic measurements. Namely, the original **mixture 3** was agitated with the catalyst in a closed vial using a Vortex shaker. In certain periods of time, the solution portions were sampled out and analyzed. The data are presented in Figure 7. With the concentration of *p*-nitrophenol at 0.1 mM, (**mixture 3**), we obtain the kinetic data (Figure 7a,b). Figure 7a represents the actual UV–Vis spectra, acquired during the experiment. It demonstrates that the peak at 400 nm, related to the reactant gradually decreases, while the peak at 300 nm, related to the product, increases. The kinetic curve, expressed as the function of ln(C_t_/C_0_) on time (Figure 7b), can be fitted by two straight lines. During the first 20 min, the slope is lower due to the induction period. After the induction period, the slope becomes higher. The reaction appears as the pseudo-first-order reaction, and the appeared rate constant is *k*_app_ = 2.61×10^−4^ s^−1^.

With the concentration of *p*-nitrophenol of 1 mM (**mixture 4**), such measurements are difficult due to the fact that the intensity of the 400 nm signal is beyond the instrument detection range (Figure 7c). However, after 696 s, there is a significant decrease in the signal intensity at 400 nm and a simultaneous appearance of the well-pronounced signal at 300 nm. After 932 s, the signal at 400 nm fully disappears, and the signal at 300 nm further intensifies. For comparison, in the literature [12], the complete reduction of *p*-nitrophenol in the 0.1 mM solution takes 4923 s. In this study, the concentration of *p*-nitrophenol is 10 times higher (1.0 mM); respectively, the quantity of the catalyst was also increased 10 times, and the reaction was complete in 932 s. Thus, based on the two experiments (Figure 6 and Figure 7), one can conclude that *r*OMC/Ni exhibits an efficiency at least comparable with that of *r*GO/Ni. At the same time, the newly developed catalyst is significantly cheaper.

In order to prove that the Ni^2+^ ions in the *r*OMC/Ni composite are responsible for its catalytic activity, we performed a control experiment with the *r*OMC sample without Ni^2+^ ions. For this purpose, the original OMC sample was reduced with sodium tetrahydroborate, according to the method described above, but without adding the Ni^2+^-containing solution. Shaking the *r*OMC sample with 1 mM *p*-nitrophenol solution and 100-fold excess of sodium tetrahydroborate (**mixture 4**) did not result in the reduction of *p*-nitrophenol (Figure S5, SI). The UV spectra show neither a decrease in the 400 nm peak intensity nor the appearance of a new peak at 300 nm for the 1st and the 2nd portions, taken after 208 s and 1275 s, respectively.

Thus, we can conclude that the coordination of Ni^2+^ ions with oxygen-containing groups imparts catalytic properties to the composite. The catalytic properties of this material are due to the high surface area of OMC and high lateral density of the active sites. The latter is explained by the highly dispersed, presumably monoatomic condition of nickel on the surface, which in turn is afforded by the oxidized nature of OMC.

The demonstrated efficiency of the newly developed catalytic system in a prototype of a continuous flow reactor suggests its applicability in real industrial applications. At the same time, this material can be prepared from inexpensive readily available reagents by simple procedures. The resulting catalyst can be used for wastewater treatment from *p*-nitrophenol. The study opens the doors for the synthesis of a new series of catalytic systems comprising metal atoms on the OMC structural support.

## 4. Conclusions

In this study, we prepared a new catalytic system comprising nickel on the OMC support. Nickel exists presumably in the form of individual ions or few-atom clusters, coordinated by the oxygen groups of OMC. The catalytic properties of the as-prepared material were tested in the reaction of reducing *p*-nitrophenol. In addition to the traditional reaction setup for this type of reaction, we also tested the catalyst in a prototype of a continuous flow reactor. Namely, the catalyst was used as a stationary phase in the column, and the *p*-nitrophenol solution was passed through the column. The catalyst demonstrated excellent catalytic efficiency; only *p*-aminophenol was registered in the outlet solution. The catalytic properties of the composite are associated with its high surface area and the high lateral density of active centers due to the atomically dispersed character of Ni on the OMC surface. The latter, in turn, is due to the coordination of the Ni^2+^ ions by the oxygen-containing groups of OMC. The main advantage of the newly developed catalytic system is its low production cost simultaneously with excellent catalytic performance. This opens the doors for its use in actual applications for wastewater treatment.

## Data Availability

Not applicable.

## Supplementary Materials

The following supporting information can be downloaded at: https://www.mdpi.com/article/10.3390/molecules27175637/s1, Figure S1: The EDX mapping of a selected area of an *r*OMC/Ni particle; Figure S2: The high magnification HAADF-STEM image from the selected area of the *r*OMC/Ni sample; Figure S3: The UV–Vis spectra of the original **mixture 1** and the first portion of its reduction product; Figure S4: The UV–Vis spectra of the original **mixture 2** and the first portion of its reduction product; Figure S5: The UV–Vis spectra for the control experiment with *r*OMC without Ni^2+^.

## Sample Availability

Samples of the compounds are not available from the authors.
