# Supplementary material for: Nickel on Oxidatively Modified Carbon as a Promising Cost-Efficient Catalyst for Reduction of P-Nitrophenol
Source: Molecules. 2022 Sep 1;27(17):5637. doi: 10.3390/molecules27175637 (PMC9458120; doi:10.3390/molecules27175637)
Supplement: Supplementary file 1 [file molecules-27-05637-s001.zip › molecules-1805956-supplementary.pdf]

Supplementary Information for

Nickel on Oxidatively Modified Carbon as a Promising Cost-Efficient Catalyst for Reduction of *P*-Nitrophenol

Shamil Galyaltdinov [1], Anna Svalova [1], Vasiliy Brusko [1], Maria Kirsanova [2] and Ayrat M. Dimiev [1,*]

[1] Laboratory for Advanced Carbon Nanomaterials, Chemical Institute, Kazan Federal University, Kremlyovskaya Str. 18, 420008 Kazan, Russia
[2] Advanced Imaging Core Facility, Skolkovo Institute of Science and Technology, 121205 Moscow, Russia
* Correspondence: amdimiev@kpfu.ru

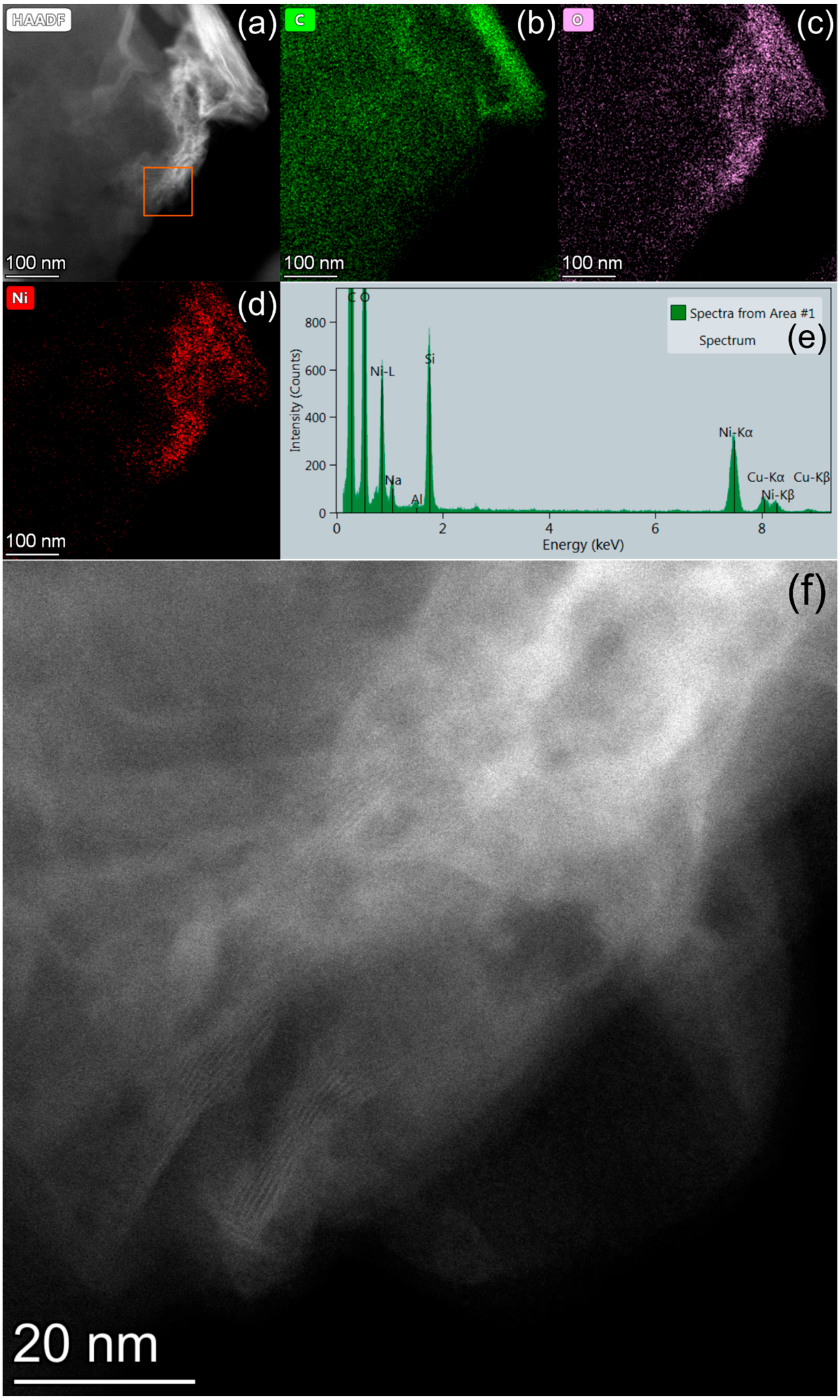

Figure S1. The EDX mapping of a selected area of an rOMC/Ni particle. (a) the HAADF image; (b) – (d) the mapping for C, O, and Ni; (e) the spectrum; (f) the higher magnification image from the area shown by yellow square on panel (a).

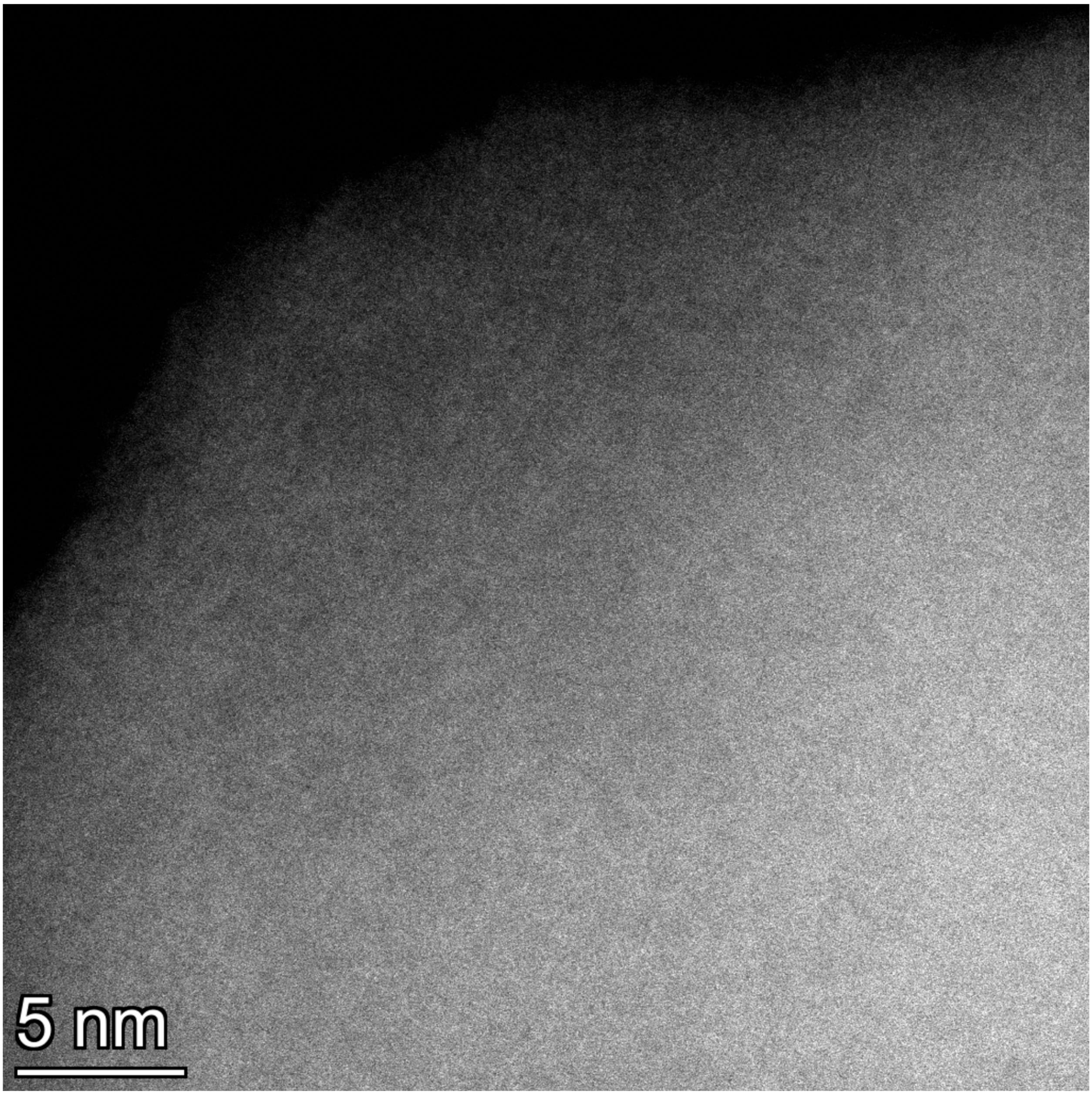

Figure S2. The high magnification HAADF-STEM image from the selected area of the rOMC/Ni sample.

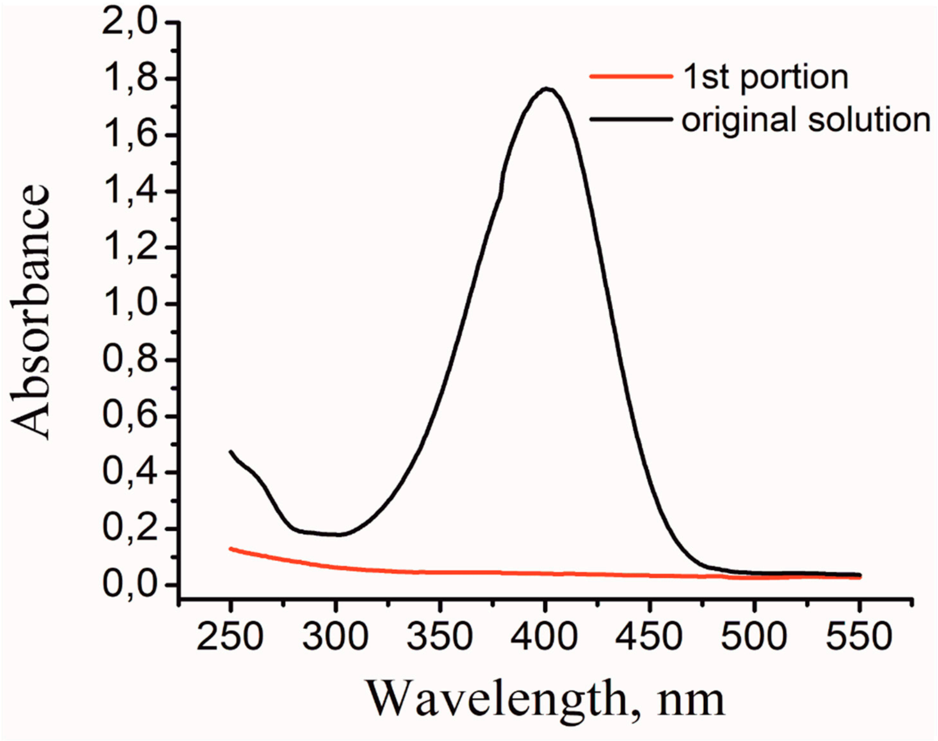

Figure S3. The UV–Vis spectra of the original **mixture 1** and the first portion of its reduction product.

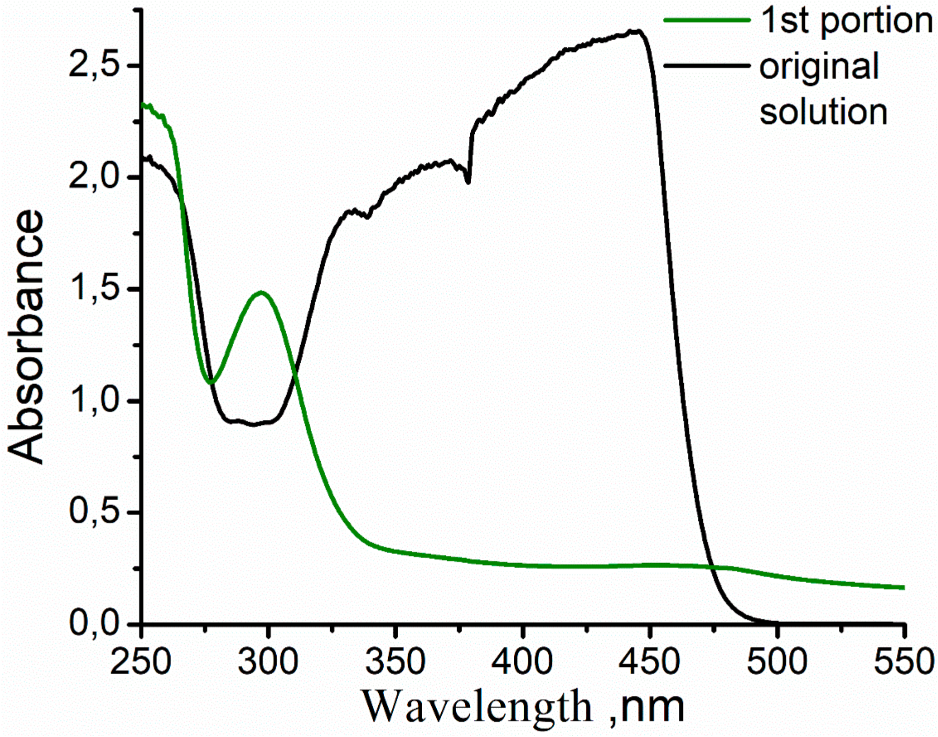

Figure S4. The UV–Vis spectra of the original **mixture 2** and the first portion of its reduction product.

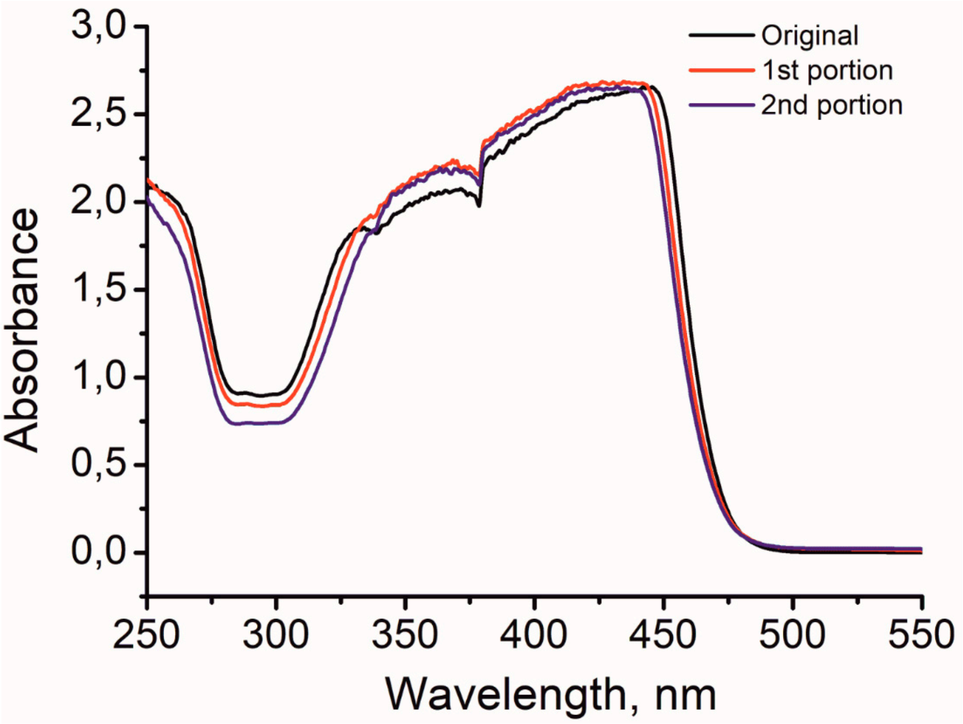

Figure S5. The UV–Vis spectra for the control experiment with *rOMC* without Ni^{2+}.
